# Randomized controlled comparison of cross-sectional survey approaches to optimize follow-up completeness in clinical studies

**DOI:** 10.1371/journal.pone.0213822

**Published:** 2019-03-18

**Authors:** Regula S. von Allmen, Christian Tinner, Jürg Schmidli, Hendrik T. Tevaearai, Florian Dick

**Affiliations:** 1 Clinics for Vascular Surgery, Kantonsspital St. Gallen, St. Gallen, Switzerland; 2 Swiss Cardiovascular Centre, Department of Cardiovascular Surgery, University Hospital Bern and University of Bern, Bern, Switzerland; Public Library of Science, UNITED KINGDOM

## Abstract

**Introduction:**

In outcome research, incomplete follow-up is a major, yet potentially correctable source of bias. Cross-sectional surveys may theoretically increase completeness of follow-up, but low response rates are reported typically. We investigated whether a pre-notification letter improved patient availability for follow-up phone interviews and thereby improved cross-sectional survey yield.

**Methods:**

A consecutive series of vascular patients was randomly divided into a trial and a validation population. The trial population was then randomized 1:1 to one of two cross-sectional contact strategies: Strategy 1 consisted of direct contact attempts by up to 12 systematically timed phone calls, whereas Strategy 2 used a personalized pre-notification letter to arrange for scheduled phone call interviews. Response rates, average time and efforts needed per patient and overall survey duration were compared. Subsequently, trial findings were externally validated in the validation population.

**Results:**

Of 728 consecutive patients, 370 were allocated to the trial population. Trial patients contacted by strategy 1 (n = 183) had a similar profile when compared to trial patients contacted by strategy 2 (n = 187). Follow-up periods following surgery (54.3 versus 53.6 months) and all-cause mortality rates (21.3% versus 18.7%) were comparable between the trial groups. Cross-sectional information on survival outcomes was almost complete after both contact strategies (99.5% versus 98.9%, *P* = 1.0). In 144/187 strategy 2 patients (77%) interviews were scheduled successfully necessitating significantly less contact attempts (median of 1.3 versus 2.3 per patient, *P*<0.0001). However, invested time per patient was similar between the groups (median of 10.1 versus 9.6 minutes), and survey strategy 1 completed earlier (median time to contact 4 versus 11 days, *P*<0.0001). Therefore, strategy 1 was validated in the validation population (n = 358): a low lost to follow-up rate below 1% (*P* = 1.0) was reconfirmed necessitating an average of 2.3 contact attempts per patient.

**Conclusions:**

Both contact strategies were equally successful in contacting almost all patients cross-sectionally. If systematically timed, direct phone calls were less complicated to organize and faster completed. Given the low time and effort per patient, outcome studies should invest in systematic follow-up surveys to minimize attrition bias.

## Introduction

Validity of outcome research depends on completeness of follow-up information [1], since incomplete follow-up is associated with the risk of missing outcome events selectively. Therefore, if two study groups differ in follow-up completeness, outcome comparisons may be flawed by this particular kind of selection bias known as attrition bias. As a consequence, follow-up information must be as complete as possible to minimise bias in outcome assessment [2, 3].

Risk of attrition bias is highest in observational studies, particularly if initiated posthoc and if follow-up assessment is based on routine clinical aftercare. However, attrition bias may theoretically also affect randomized controlled trials since the pre-interventional randomization process can neither balance out nor preclude differences arising during outcome assessment.

Cross-sectional surveys are a possible method to obtain complete outcome information per a given time point [1]. Depending on the study endpoint questionnaire surveys, phone interviews and outpatient visits can theoretically be used. Questionnaires are least expensive but carry a considerable risk of low response rates (i.e. selection bias) as well as information bias [4]. In addition, they are confined to information that patients could be expected to self-report in an accurate manner. Outpatient visits, on the other side of the spectrum, offer the advantage that outcomes may be ascertained by trained individuals, but they are impractical for many reasons including the impossibility to assess all patients simultaneously and that these cost-intensive visits are rarely reimbursed if not driven clinically.

Standardized phone interviews have a number of advantages: they provide a direct patient contact and are suitable for assessment of many endpoints. However, they are deemed time consuming and may be frustrating if contact numbers are incorrect or patients cannot be reached quickly. Even if successful, unexpected calls may come as a surprise to the patient carrying the risk of recall and interviewer bias [5].

There is evidence that combining tracing methods may improve success of contact [6]. We hypothesized that an advance notice might facilitate organization of telephone interviews in terms of patient availability and disposition. Thus, this study assessed whether in cross-sectional surveys a pre-notification letter to organize a phone interview improved efficacy of telephone surveys while reducing typical disadvantages.

## Materials and methods

This randomized controlled trial compared two distinct cross-sectional follow-up survey strategies (contact strategy 1 *versus* contact strategy 2, see below) in randomly allocated patients. Patients who had undergone open or endovascular abdominal aortic aneurysm (AAA) repair between June 2001 and December 2010 at the University Hospital of Bern, Switzerland, were eligible. They were identified from a prospective registry, and consecutive completeness was cross-checked against all surgical records from the same time period [1]. Patients already known to be dead were excluded, whereas assumed survivors were randomly divided 1:1 into a trial and a validation population. The trial population was then divided again in a pragmatic 1:1 randomized, parallel-group study design with groups being allocated either to contact strategy 1 or 2. Depending on trial outcome, the preferred strategy was to be validated within the validation population.

All patients had formally consented in writing at the time of surgery to be contacted during follow-up and for anonymized analyses (informed consent). This written consent to research participation had been discussed and reconfirmed at every outpatient follow-up contact. No posthoc changes were applied to the survey methods after the commencement of the study.

The present health research was deemed purely methodological and included no therapeutic intervention in participants; therefore, the trial was not pre-registered. Study design and analysis plan were approved by the cantonal research ethics committee Berne, Switzerland. All data were anonymized before analysis.

### Investigated contact strategies

**Fig 1:**
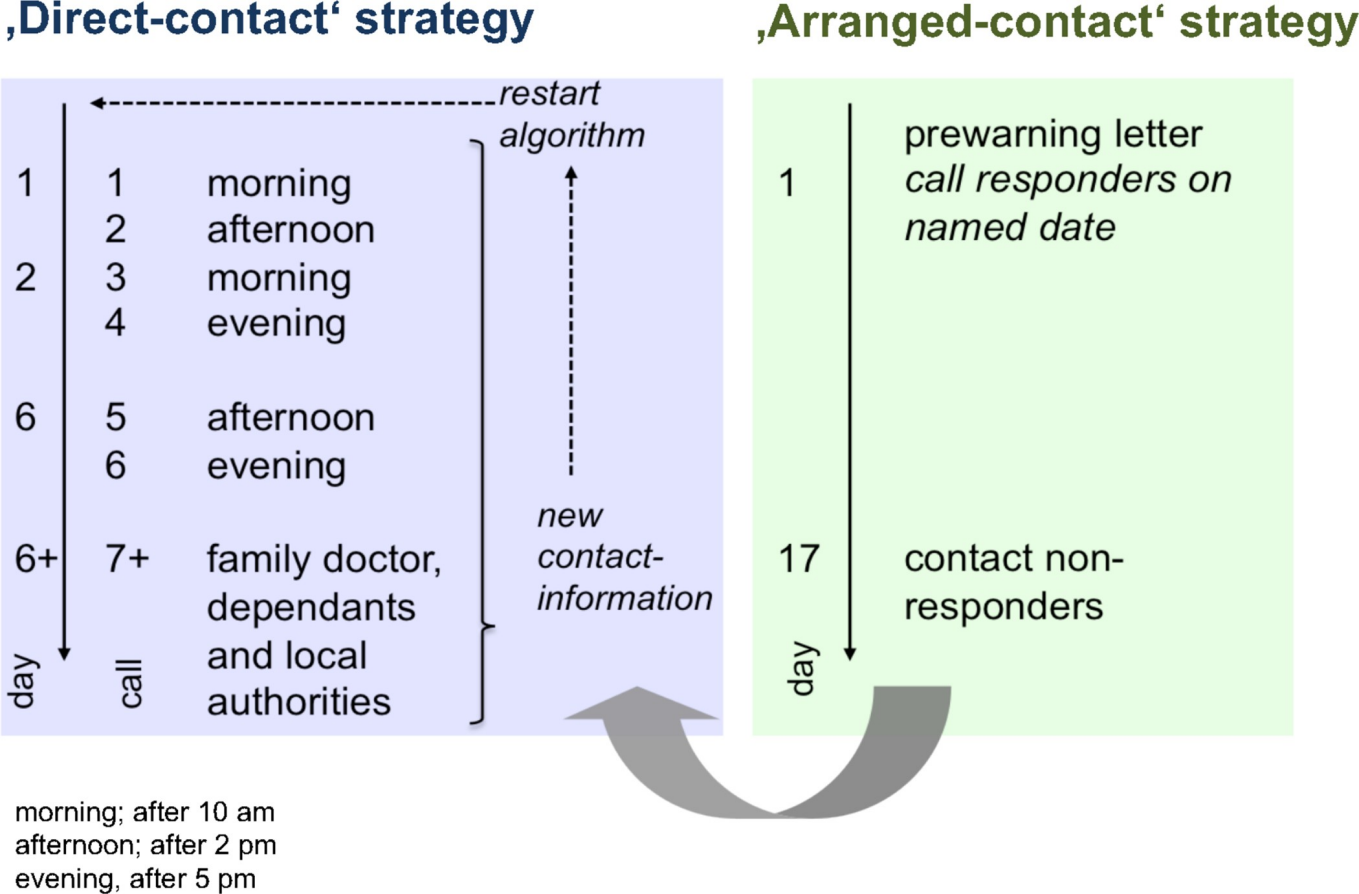
A scheme of the applied contract strategies. Fig 1 outlines the predefined contact schedule for the ‘direct contact’ (contact strategy 1) and the ‘arranged contact’ (contact strategy 2) group.

The contact strategies were applied by the same investigator (CT): Contact strategy 1 consisted of unannounced phone calls using the last registered phone number (Hospital administrative database; Systems, Applications and Products, SAP, Walldorf, Germany). A maximum of six contact attempts followed a predefined schedule (Fig 1). If attempts were not successful, general practitioners and/or designated relatives were contacted to confirm if this patient was still alive and whether the phone number had changed. Then the contact algorithm was restarted using any updated contact details. Thus, the number of contact attempts was limited to a theoretical maximum of twelve per patient. As last resort, municipal administrations were inquired. Patients who could not be traced in any way were categorized ‘lost to follow-up’.

Contact strategy 2, in contrast, sent personally signed letters to all participants first using the last registered address in the hospital administrative database. These letters carried official insignia including the phone number of the research office and explained the rationale behind the present investigation. It also asked for reconfirmation of the registered phone number and for a convenient time for the telephone interview within a small range of the predefined study end date. An addressed and stamped envelope was attached for response. Responses were registered and interviews scheduled at the indicated times. A latency of 16 days was accepted before non-respondents were contacted according to the ‘direct contact’ strategy. Responders who refused the interview were not contacted but included for survival analysis if the response came from the patients themselves.

#### Structured interviews

Patient interviews were conducted in one of the three main Swiss languages (i.e. German, French or Italian) and were all led by the same investigator (CT). A standardised structure including the following 4 sets of standardized questions was used: (1) were the patients able to recollect the aortic operation and type of repair (open versus endovascular)? (2) how were present health state and physical fitness (using standardized categories, see below)? (3) what was the degree of independency in daily living? And (4) had patients undergone any intervention since the operation (either aneurysm-related or not)? (**S1 and S2 Files)**

Based on the patients’ self-assessment, the subjective current overall health condition was categorized according to a Likert scale ranging from “excellent” over “well” and “fair” to “poor”. Physical fitness was measured by estimating maximum metabolic equivalents of task (MET), which express the energy cost for specific physical activities in kcal/kg/hour. Typical MET values range from 0.9 (sleeping) to 23 (running at 22.5 km/h pace). The compendium of physical activities [7] was used for standardized MET assessment. Independency was categorized into “autonomous living”, “needing support from relatives only”, “depending on mobile nursing services” or “living in a nursing home”.

### Baseline and outcome measures

Baseline patient characteristics included conventional demographics, date and type of initial aortic operation and follow-up period until the study survey.

Primary outcome was the proportion of verified survival information at cross-sectional survey (i.e., patient either alive or registered date of death versus patient being lost to follow-up). Dates of death were ascertained either by information from patients’ relatives or municipal administrations. Kaplan-Meier curves were used to estimate cumulative survival rates based on this information.

Secondary outcome measures included time and effort invested per survey-strategy (ie., number of phone calls, cumulative work time per patient, and time period between start of the survey and the actual interview). Work time estimates per patient were given in minutes and included (1) average time needed per interview (cumulative time spent for interviews divided by the number of patients) and (2) the overall time invested per individual patient. The latter summed up all contact attempts including those to relatives and general practitioners. For contact strategy 2 an equal share of the 90 minutes needed to draft the invitation letter was added to every patient plus an additional 3 min for dispatching the letters.

The ‘elapsed time until interview’ was measured in days and per patient: for contact strategy 1, it started for all patients at the day when contact attempts started and counted the days until successful individual phone interviews. For contact strategy 2, in contrast, it started with the dispatch of the invitation letters, which were all sent out at the same day.

### Analysis plan

All analyses were predefined, and the analysis plan was agreed before data were inspected. Baseline information was retrieved electronically from the prospective patient registry. Outcomes were collected on paper during the interviews. All data were subsequently anonymized and transferred into a dedicated database (Microsoft Access. Redmond, Washington). Analyses used IBM SPSS statistics (version 21.0 for Windows, IBM Corporation, Software Group, Somers, New York). **(S3 File)**

To detect a minimum difference in follow-up completeness of 15% (72% versus 87% based on previous study findings [8]) at a power of 90% and at a 5% alpha level, the required minimum overall sample size was 300 (sample size calculator at clincalc.com) [9]. Adding a safety margin of 20% (anticipated fraction of patients already known to be dead in the patient population) the target size of the trial population was determined at 360.

Random allocation sequences (both for inclusion into either trial or validation population, and within the trial population for assignment to one of two contact strategies, respectively) were generated centrally using a freely available computer-based software (www.randomizer.org). No restrictions were implemented to the type of randomization. The investigator performing the survey (CT) could not be blinded for obvious reasons; neither could the patients who however, were not aware of the trial before being contacted within the allocated strategy. In contrast, the investigator performing the statistical analyses (RvA) was blinded towards group allocation. The contact strategy with the superior outcome regarding follow-up completeness and speed of survey completion was to be validated externally in the validation group.

#### Statistical methods

Endpoints were analyzed according to intention to treat. Assuming an efficient randomization process, comparisons were not adjusted for confounding factors; neither in the trial, nor during validation. Patient characteristics are described using conventional summary statistics. Continuous variables were not assumed normally distributed and therefore compared using Mann-Whitney-U test. Proportions were compared using Fisher’s exact test. Median duration of the survey was estimated using Kaplan Meier curves, and time to survey completion was compared using log rank-test. Validation results were compared with the trial findings analogously. All tests were two-sided, and an alpha level of 0.05 was chosen to assume statistical significance of differences.

## Results

**Fig 2:**
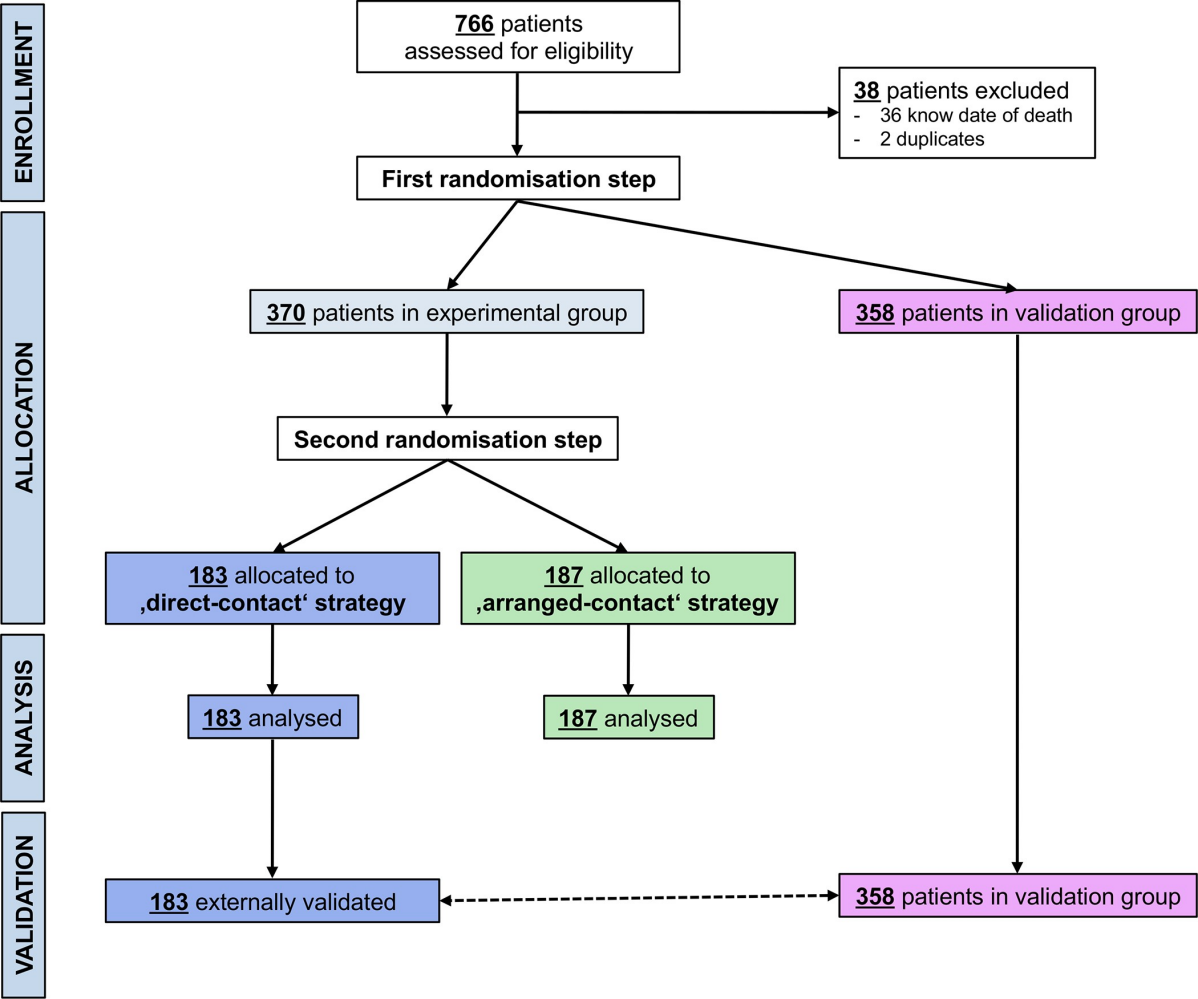
CONSORT diagram. The CONSORT diagram shows the patient flow through the study including trial and external validation.

The patient flow through the whole study including both, trial and subsequent validation, is detailed in the CONSORT diagram (Fig 2). In brief, 766 patients were potentially eligible; 38 were excluded either because they were already known to be dead or because they were duplicate entries in the database. Thus, 728 participants were included: 370 were randomly allocated to the trial population and 358 to the validation population. Within the trial population, 183 participants were randomly assigned to the contact strategy 1, and 187 to the contact strategy 2. No participant was excluded after group allocation. The whole trial population was contacted between 21^st^ February 2011 and 10^th^ March 2011 using the allocated strategy, and all were analyzed according to intention to treat. Secondary outcomes were obviously assessed only in surviving participants who could be contacted. The validation process (n = 358) took place between 14^th^ March 2011 and 24^th^ March 2011.

### Baseline patient characteristics

Table 1 presents the baseline characteristics of both trial groups as well as the validation population. Baseline characteristics were very similar across all study groups: Median age was 76 years (interquartile range (IQR), 69–82), 90.5% were men, and the median follow-up period between aortic intervention and the present cross-sectional study was 53.9 months (IQR 31–80). This implies successful randomization and, therefore, comparable patient groups regarding the included potential confounding factors.

**Table 1:** Baseline characteristics as well as trial and validation outcomes.

		Stategy 1 (n = 183)	Strategy 2 (n = 187)	Validation of strategy 1 (n = 358)
**Demographic characteristics**				
	Median Age, *years (IQR*^*a*^*)*	76 (69; 82)	75 (68; 82)	75 (68; 81)
	Male patients, *n (%)*	(91.3%)	(90.4%)	328 (91.1%)
	Median time since operation, *months (IQR*^*a*^*)*	54.3 (31.4; 91.7)	53.6 (29.6; 77.2)	55.5 (29.7; 85.9)
**Trial/validation findings: Survival, physical fitness and independency**				
	Patients alive, *n (%)*	143 (78.1%)	150 (80.2%)	279 (77.9%)
	Patients died during follow up, *n (%)*	39 (21.3%)	35 (18.7%)	77 (21.5%)
	Lost to follow up, *n*	1 (0.5%)	2 (1.1%)	2 (0.6%)
	Median MET^b^ *(IQR*^*a*^*)*	4 (3, 5)	4 (3, 4)	4 (4; 5)
	Independency			
	- Provided no information on independency, *n (%)*	3 (1.6%)	2 (1.1%)	10 (3.6%)
	*-* Autonomous living, *n (%)*	116 (81.1%)	126 (84.0%)	223 (79.9%)
	*-* Needing support from relatives only, *n (%)*	11 (7.7%)	9 (6.0%)	25 (9.0%)
	- Depending on mobile nursing services, *n (%)*	10 (7.0%)	6 (4.0%)	10 (3.6%)
	*-* Living in a nursing home, *n (%)*	3 (2.1%)	7 (4.7%)	11 (3.9%)

^a^IQR; interquartile range

^b^MET = metabolic equivalent of task

### Trial outcomes

Table 1 summarizes the trial findings. Overall, cross-sectional survival status was ascertained in 367/370 patients corresponding to completeness of follow-up information at 99.2%. In the strategy 1 group, one patient (out of 183, 0.5%) was deemed lost to follow-up, and in the strategy 2 group there were two (out of 187, 1.1%, *P* = 1). An official date of death was registered for 39 patients and 35 patients, respectively, leading to a similar overall mortality between the groups (21.3% versus 18.7%, *P* = 0.702). Accordingly, 143 strategy 1 patients were alive at the time of the survey (3 refusing the detailed interview), and 150 strategy 2 patients (2 refusing the interview).

In the strategy 2 group, significantly more patients were able to recollect details of the operation (98% versus 93% in the strategy 1 group, *P* = 0.046). However overall, only 9 out of 10 of those participants were actually correct when describing aortic intervention (92.6% versus 88.5%, *P* = 0.478): 16 patients confused type of operation (n = 7 versus n = 9) and 11 were unable to provide any information regarding type of repair (n = 4 versus n = 7).

Similar proportions of survivors felt completely recovered from the operation in both trial groups (94.2% vs 94.0%, *P* = 1.0) and most considered themselves in good or excellent health condition (84.6% versus 90.7%, *P* = 0.270). Also, current physical capacity and independency were similar between the groups: the overall median was 4 MET (IQR 3–5, Table 1) and similar proportions lived autonomously (81.1% versus 84.0%, *P* = 0.632). Only a minority lived in a nursing home (2.1% versus 4.7%, *P* = 0.337). The remaining patients were supported either by relatives or by a mobile nursing service. (Table 1)

#### Duration of the survey

**Fig 3:**
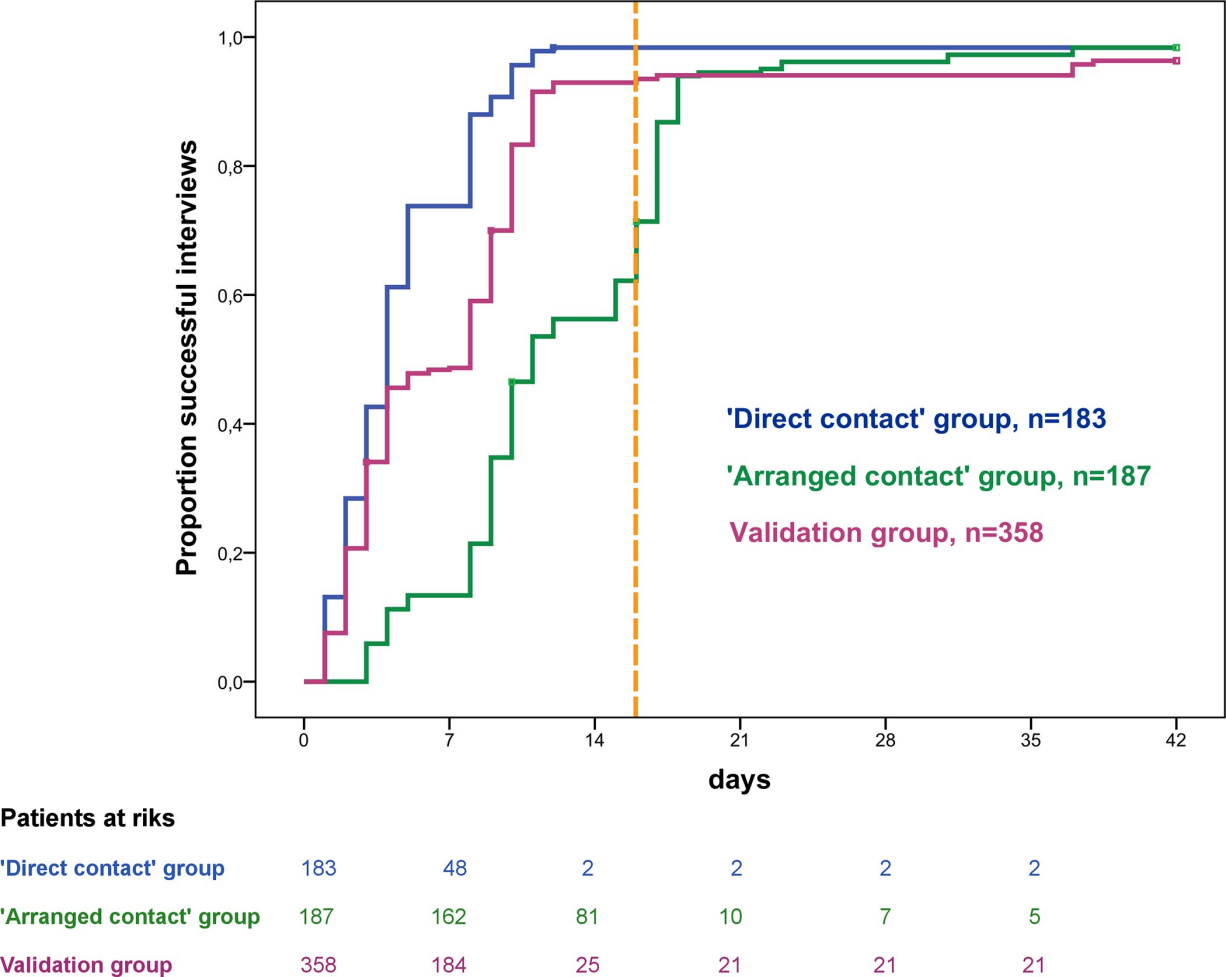
Time to successful interview. Fig 3 shows cumulative Kaplan Meier estimates of the proportion of successful interviews over time. Data are stratified for the three groups: strategy 1 versus strategy 2 versus validation of strategy 1. The vertical dashed orange line marks the allowed pre-set latency of 16 days in the ‚arranged contact’ group (contact strategy 2) to await participants’ responses. The ‘direct contact’ strategy (contact strategy 1) succeeded with a shorter median duration to successful interview and was then re-evaluated in the validation group showing a similar duration to successful interview.

Contact strategy 1 succeeded with a shorter median duration to successful interview (4 days (IQR 2–8) versus 11 days (IQR 9–17), *P*<0.001), but this difference was influenced by the pre-set latency of 16 days to await participant responses in the contact strategy 2 group (Fig 3). A total of 144 letters were returned spontaneously (77%), 137 (95%) of which were returned within the predefined latency. In 23 cases no subsequent interview was scheduled (2 patients refused an interview and 21 response letters provided an official date of death). The remaining 43 non-responders (23%) were contacted using the ‘direct contact’ strategy.

#### Number of phone calls

A total of 414 calls was needed for contact strategy 1 compared with 246 for contact strategy 2. This corresponds to an average of 2.26 (IQR 1; 3) phone calls per patient in contact strategy 1 versus 1.32 (IQR 1; 2, *P*<0.001) in contact strategy 2, respectively.

#### Invested work

In absolute terms, 30 hours and 44 minutes were invested to contact the 183 patients in the contact strategy 1 group, while 30 hours and 8 minutes were invested to contact the 187 patients in the contact strategy 2 group, which included a 90 minutes surcharge for drafting and 3 min per patient for dispatching the invitation letters. As a consequence, an average of 10.1 versus 9.6 minutes was needed per patient.

### Validation

The equally effective, but less complicated (no scheduled arrangements) and overall faster strategy (i.e. strategy 1) was re-evaluated within the validation population (n = 358). Baseline characteristics and overall mortality following the operation were similar as in both trial groups (Table 1). Primary outcome did not differ either; the lost to follow-up rate was 0.6% (n = 2; versus one in the trial contact strategy 1 group, *P =* 1.0) and an average of 2.34 call attempts per patient was required which was also similar to the trial group (2.26 attempts per patient, *P* = 0.795).

## Discussion

This study investigated and validated relatively inexpensive and readily available methods to assess outcome information in cross-sectional surveys within clinical outcome research. The main and somewhat surprising finding was not the absence of relevant differences between the investigated contact strategies, but that successful and reliable tracing of more than 98% of patients in relatively large study populations was reproducibly possible within a few days of a predefined study end date and with an investigator investment of less than 15 minutes per patient. Although the contact strategy relying on a pre-notification letter (contact strategy 2) necessitated only half of contact attempts, it neither reduced overall investment of time and effort nor did it improve follow-up completeness. In contrast, it increased procedural complexity (sending the letters, monitoring of returns, scheduling the phone interviews according to patient preferences and tracing non-responders) and delayed completion of the survey due to the dispatch of the letter. Thus, the hypothesis of our study was not reconfirmed but the overall goal of a complete cross-sectional follow-up was reached.

Completeness of follow up is critical because attrition bias can severely compromise internal and external validity of study findings. It therefore needs to be measured and declared, for instance by using the follow-up index [1]. As a rule of thumb it is considered that <5% loss of patients during follow up leads to little bias, while >20% poses serious threats to validity, with even less than 20% of incomplete follow up data being a problem [10]. The risk of bias however is relative and depends on the investigated endpoint and population. For instance, missing follow-up information regarding survival may affect outcome analyses more in frail patient populations undergoing major surgery (e.g. patients undergoing AAA repair) than in a younger and healthy patient cohort undergoing hernia repair.

Hard endpoints such as survival or subsequent operations usually represent the central outcome measures in observational studies and can usually be assessed without inviting the patient back to the outpatient clinic. Completeness of follow-up is best assured by one of three ways. Prospective studies may implement a strictly scheduled follow-up surveillance (e.g. at 90 days, 1 and 3 years), which carries the advantage of equal follow-up periods for each patient allowing for calculation of absolute outcome percentages at the predefined intervals. Although this approach provides the most precise and reliable outcome estimates, it is work-intensive, expensive and can rarely be implemented in clinical routine. In addition, it is only possible in truly prospective research projects and restriction to identical follow-up periods in all patients leads to loss of large proportions of potentially available follow-up information.

Alternatively patient samples may be cross-referenced with official and up to date registries of death or interventions. This provides the most reliable, accessible and complete follow-up information if all available patient information is to be used, but it provides only cumulative outcome estimates (e.g. Kaplan Meier) and it is not available in most countries without a national health service.

The last alternative is a comprehensive cross-sectional survey of the whole study population at a predefined study end date, such as in the present study. Similarly to the use of official registries this approach accepts variable follow-up periods for cumulative Kaplan Meier outcome estimates, but researchers need to ensure up to date completeness of cross-sectional information [1]. This challenge is often avoided because of assumed impracticability and fear of failure, but also because lack of follow-up completeness may easily pass unnoticed and the risk of attrition bias is underestimated [1]. This study demonstrated that it is possible and worth taking on the challenges of comprehensive cross-sectional surveys even in larger patient populations.

Regardless of the method, used return rates are crucial for survey validity particularly if the reasons for (non)-responding may be causally linked to any of the collected outcome information (e.g. deceased patients cannot respond) [11].

In postal questionnaire surveys, for instance, return rates usually range around 50 to 65%, depending on patient demographics, provision of stamped return letters and bond between patients and health care providers [12]. The same applies to typical contact rates in phone survey [13].

Several approaches have been explored to increase survey completeness. In a Norwegian study assessing 633 patients after surgery for degenerative disorders of the lumbar spine, some 78% responded to the initial postal questionnaire. Using systematic efforts to trace non-responders eventually resulted in a similar overall contact success as the present study, even if not at the same speed. Only 2.8% had to be classified as lost to follow up [14].

Another approach to increase contact success consists of combining tracing methods, for instance by notifying participants of a planned survey before the actual contact attempt. However, scientific evidence of whether a pre-notification letter is beneficial in health research is conflicting. In smaller scale studies and two small systematic reviews, a pre-notification letter increased response rates considerably [11, 12, 15], whereas it had no impact on response rates in a more recent larger scale study[16]. At least, respondents who received a pre-notification letter were inclined to respond earlier [16].

Previous studies also assessed alternative strategies to increase response rates to mailed questionnaires. For instance the odds of a response increased if a monetary incentive was used [17], if the letter was signed individually and if a hand-addressed postage-paid return envelope was added [18, 19]. The present trial intervention (contact strategy 2) implemented several of these approaches. The notification letter was signed individually containing a postage-paid return envelope, and it was participant-friendly with easy-to-follow instructions asking only very few questions, such as the preferred date and time for an interview and the current phone number. Subsequently, it achieved an above-average early response rate of 77%. In the present setting, however, contact strategy 1 yielded comparable contact rates.

This suggests that the study findings did not depend on particular aspects of any contact strategy but were driven by the systematic overall approach. Both strategies used structured contact sequences, starting by approaching patients via systematically timed phone calls, contacting relatives and general practitioners and finally contacting municipal authorities. Such a structured contact sequence is particularly important in an elderly population in which many patients may have moved to a nursing home during the follow up time. In our present study, some 3% of the patients lived in a nursing home at the time of the interview. Such highly selected and probably most vulnerable patient subgroups should not be missed in cross-sectional surveys to rule out selection bias. Assumedly, the preferred contact algorithm of a cross-sectional survey may be distinct in populations with different demographics.

Although the pre-notification letter failed to improve contact strategy 1, a potential benefit lies in better informed patients at the time of the actual interview [20]. This advantage may be accentuated if the interview is complex and asks for detailed information. Indeed, in the present study, patients receiving a pre-notification recollected the type of aortic operation significantly better than patients who were surprised by the direct phone call; however the information advantage was not reflected in a shorter individual interview time or a higher degree of accuracy of the remembered information.

A disadvantage of a notification letter is the potential prolongation of the overall survey time, since dispatch of such letters and processing of the brief questionnaire may be associated with a delay. Therefore, it is not surprising that contact strategy 1 completed the survey significantly earlier, although it did not reduce the average time invested per patient. Also, the efficiency of strategy 1 probably depends on the availability of the interviewer; in contrast, contact strategy 2 allows the investigator efforts to be concentrated to scheduled interview slots.

Nowadays, many people are used to other forms of communication, such as short messenger service (SMS), email or web-based electronic surveys. The effect of a SMS text notification on the response rate to a postal questionnaire was investigated in a randomized controlled trial. The authors found no impact of SMS on response rates, although there was a notion that it might be effective in female participants [21]. Electronic surveys carry some obvious advantages over other types of surveys; they are less costly, faster applied and more flexible than postal or phone surveys, but they are also associated with significant disadvantages [22]. Generally, electronic surveys had lower response rates compared to more traditional survey methods [23]. A successful completion of an electronic survey also relies on specific prerequisites, such as availability of an accurate up-to-date and complete email address list for the participants. This may be particularly relevant in an elderly group of participants, as in the present study, since elderly people are less familiar with electronic communication. Many do still not have an email address or computer and thus may not be contacted electronically [24].

The present study has particular strengths and limitations. The main strength of the study lies in its experimental design. Due to randomization, the groups being investigated were highly comparable with a low risk of confounding. The survey was conducted by a single investigator according to a predefined contact algorithm, which reduced any bias associated with interobserver variability. Moreover, a formal validation was performed in external patients to reconfirm the trial findings. All of this suggests a high degree of internal and external validity of study findings. The main study limitation may be seen in that it failed to link any of the investigated interventions causally to cross-sectional survey success. This is most likely due to the systematic structure of both approaches, but may also be influenced by the investigated study population which consisted of elderly and typical vascular patients, living in a middle European society where health service insurance is mandatory and the level of social security is high. Therefore our findings may not be generalized to populations with different ethnic, socioeconomic or demographic characteristics. For instance, it is possible that the same trial in a younger (working) patient population would have led to different findings. In such a setting, the preferred strategy might well have been to schedule interviews via a pre-notification letter. However, the Norwegian study that assessed the behaviour to postal questionnaires among patients operated for degenerative spine disorders has shown that particularly younger patients are less likely to respond to postal surveys [14]. As a consequence, one may assume that a systematically timed algorithm with various contact attempts at different daytimes may compensate for demographic disparities among different patient populations and that such a strategy may be applied for many types of postoperative follow up surveys.

### Conclusion

Incomplete follow-up and low response rates to cross-sectional outcome assessments should not be accepted as an intrinsic defect of outcome studies. Both investigated contact strategies were equally successful in contacting almost all patients cross-sectionally. If systematically timed, direct phone calls were less complicated to organize and completed faster. Given the low time and effort per patient, outcome studies should invest in systematic cross-sectional follow-up surveys to minimize risk of attrition bias.

## Supporting information

S1 File S1 File shows the standardized telephone survey questionnaire in English that was used for the interview. (PDF)

S2 File S2 File shows the standardized telephone survey questionnaire in German that was used for the interview. (PDF)

S3 File S3 File contains the collected baseline and outcome data. (XLSX)
