# Supplementary material for: Randomized controlled comparison of cross-sectional survey approaches to optimize follow-up completeness in clinical studies
Source: PLoS One. 2019 Mar 18;14(3):e0213822. doi: 10.1371/journal.pone.0213822 (PMC6422260; doi:10.1371/journal.pone.0213822)
Supplement: S1 File — (PDF) [file pone.0213822.s001.pdf]

1. Contact attempts and general patient information

1.	2.	3.
4.	5.	6.
relatives	Fam. doctor	Local government

Patient identification number

Name of patient

Date of birth:

Phone number:

Family doctor:

Hospitalised: *date*

Date of surgery: *date*

Type of surgery	open □	EVAR □
Patient or relatives reached?	yes □	no □
Patient died?	yes □	no □
If yes, date of death: ………………….		
Patient moved?	yes □	no □

2. Assessment of recollection of aortic operation

The „opener“:

- Do you remember your surgery? yes □ no □

3. Assessment of patient’s health status

Health status:

- Did you recover from surgery? yes □ no □
- Do you feel still affected of the surgery or its consequences? yes □ no □

How would you describe your general health status?

- excellent 1
- very good 2
- poor 3
- very poor 4

Comments: Why is the general status good / poor?
Performance:

4. Assessment of metabolic aequivalent of task (MET)

**Question**	**MET**
Can you live independent in your apartment?	2
Can you walk independent 1-2 blocks?	3
Can you clean up?	3
Can you climb stairs one floor up or walk upwards?	4
Can you run a short distance?	5
Can you cycle?	6
Can you ski?	7

MET
<3: light
3-6: moderate
>6: vigorous

5. Assessment of degree of independency

- Can you look after yourself independently? yes □ no □
- Do you need support of home cae? yes □ no □
- Do you live in a rest/nursing home? yes □ no □

6. Assessment of re-interventions and re-hospitalisations since index

- Did you have a second surgery caused of your aorta? yes □ no □

- If „yes“ when? - date of re-surgery:
- If „yes“ why ?

- Have you been rehospitalised since your first aortic surgery?
  yes □ no □

- If „yes“ when? - date:
- If „yes“ why ?

---

7. Completeness of questionnaire

Questionnaire complete ? yes □ no □
