# Supplementary material for: Randomized controlled comparison of cross-sectional survey approaches to optimize follow-up completeness in clinical studies
Source: PLoS One. 2019 Mar 18;14(3):e0213822. doi: 10.1371/journal.pone.0213822 (PMC6422260; doi:10.1371/journal.pone.0213822)
Supplement: S2 File — (PDF) [file pone.0213822.s002.pdf]

1. Kontaktversuche und generelle Patienteninformation

1.	2.	3.
4.	5.	6.
Angehörige	Hausarzt	Gemeindeverwaltung

Patientenidentifikationsnummer

Pateintenname

Geburtsdatum

Telefonnumner

Hausarzt

Hospitalisation: *Datum*

Operation: *Datum*

Operationsart	offen □	EVAR □
Patient oder Angehörige erreichte?	Ja □	Nein □
Patinent verstorben	Ja □	Nein □
Wenn ja, Todesdatum …………………		
Patient verzogen?	Ja □	Nein □

2. Beurteilung des Erinnerungsvermögens

Der „opener“:

- Können Sie sich an den Eingriff erinnern? Ja □ Nein □

3. Assessment of patient’s health status

Health status:

- Haben Sie sich von dem Eingriff gut erhol Ja □ Nein □
- Fühlen Sie Sich durch den Eingriff und dessen Folgen heute beeinträchtigt?
  Ja □ Nein □

Wie würden Sie Ihren Gesundheitszustand im Allgemeinen beschreiben?

- Ausgezeichnet 1
- Sehr gut 2
- Weniger gut 3
- Schlecht 4

Comments: Warum ist der Allgemeinzustand schlecht / gut?

Leistungsfähigkeit:

4. Erhebung der metabolic aequivalent of task (MET)

Frage	MET
Können Sie sich alleine in der Wohnung bewegen	2
Können Sie selbständig am Stück 1-2 Wohnblöcke weit gehen	3
Können Sie den Abwasch machen	3
Können Sie eine Treppenetage oder bergan gehen	4
Können Sie eine kurze Distanz rennen	5
Können Sie noch Radfahren (langsam)	6
Können Sie noch Skifahren	7

MET
<3: light
3-6: moderate
>6: vigorous

5. Erhebung der Selbständigkeit

- Können Sie sich eigenständig versorgen ? Ja □ Nein □
- Benötigen Sie „Spitexbetreuung“ ? Ja □ Nein □
- Leben Sie in einem Pflege-/Altersheim ? Ja □ Nein □

6. Erhebung der Re-Operationen oder Re-Hospitalisationen nach der Aortenoperation

- Sind Sie seit dem Eingriff an der Bauchschlagader nochmals operiert worden ?
  Ja □ Nein □

- Wenn „ja“, wann ? Datum Re-Operation eintragen:
- Wenn „ja“, weshalb ?

- Sind Sie seit dem Eingriff an der Bauchschlagader nochmals im Spital aufgenommen worden ?

  Ja □ Nein □

- Wenn „ja", wann ? Datum Re-Hospitatlisation eintragen:
- Wenn „ja", weshalb ?

---

7. Ist der Fragebogen komplett ausgefüllt

Fragebogen komplett ? Ja □ Nein □
